# Comparative clinical performance of stainless steel, zirconia, and Bioflx crowns in primary molars: a randomized controlled trial

**DOI:** 10.1186/s12903-025-05869-8

**Published:** 2025-04-18

**Authors:** Ahmed Abdelhafez, Vineet Dhar

**Affiliations:** 1 Delta University for Science and Technology, Gamasa, Egypt; 2 Department of Orthodontics & Pediatric Dentistry, school of Dentistry, University of Maryland, Baltimore, Baltimore, United States

**Keywords:** Stainless steel crown, Zirconia crown, Bioflx crown, Primary molars, Plaque accumulation, Gingival health, Crown retention, Clinical performance

## Abstract

**Background:**

Choosing the appropriate crown type for primary molars is essential for effective restoration and oral health. Stainless steel crowns are durable and cost-effective, making them suitable for extensive decay. Zirconia crowns offer esthetic advantages, while Bioflx crowns require less preparation and provide optimal appearance. Understanding the benefits and limitations of each type ensures optimal outcomes for young patients. The purpose of the study was to clinically compare these three types of crowns for primary molars regarding plaque accumulation, debonding rate, crown substance loss and gingival health.

**Materials and methods:**

Registered with clinicalTrials.gov (NCT06706167), this study included 75 children (43 males, 32 females; mean age: 6.3 years) divided into 3 groups: Group A (stainless steel crowns), Group B (zirconia crowns), and Group C (Bioflx crowns), with 25 patients each. Plaque index, crown survival regarding debonding rate and substance loss, and gingival index were evaluated at baseline, 6, and 12 months.

**Results:**

At 6- and 12-month follow-ups, no significant differences were observed among groups. However, zirconia crown demonstrated better results in terms of plaque accumulation and gingival health. Conversely, stainless steel crown showed marginally better performance in crown retention.

**Conclusion:**

All crowns showed acceptable clinical performance. Factors such as crown retention, esthetics and biocompatibility should be considered when selecting the most appropriate crown for each molar.

**Trial registration:**

The study was registered on ClinicalTrials.gov, with the registration number NCT06706167 with registration date 26,112,024.

**Supplementary Information:**

The online version contains supplementary material available at 10.1186/s12903-025-05869-8.

## Introduction

Dental caries is a highly prevalent condition, particularly among young children [1]. Deep carious lesions are the most common issues affecting primary teeth, with 42% of children aged 2 to 11 experiencing dental caries, averaging 1.6 decayed teeth per child [2]. Given their essential roles in chewing, speaking, and serving as natural space maintainers in the dental arch, decayed primary teeth require prompt intervention [3]. Maintaining the health of primary dentition has always been vital for supporting children’s dental development [4]. In today’s conservative dentistry approach, there is a greater emphasis on repair rather than replacement [5]. 

However, managing dental caries in children has been a persistent challenge [3]. Difficulties such as obtaining cooperation from young patients and ensuring parental satisfaction complicate the treatment of severely damaged primary teeth for pediatric dentists. Moreover, the durability of restorations is crucial, as it directly impacts the preservation of tooth structure until natural exfoliation occurs [4]. 

To address these challenges, various full-coverage restorations have been developed and adopted in pediatric dentistry. Among these, stainless steel crowns (SSCs) have emerged as the most popular and widely accepted option for both primary and young permanent teeth. For more than 50 years, SSCs have demonstrated greater durability and longevity compared to other materials such as amalgam and composite. When temporary full-coronal coverage is required, no other restorative material matches the affordability, reliability, and durability of SSCs in pediatric dentistry [6]. 

Previous research indicates that SSCs have a success rate of up to 97.2%, making them a preferred restoration choice for young children at high risk for caries [4]. However, one significant drawback of SSCs is their metallic appearance, which can be unappealing to both children and their parents. Despite their numerous advantages, some professionals remain hesitant to use SSCs due to parental concerns regarding esthetics. Nonetheless, they are generally easier to place than intracoronal restorations and offer significantly better long-term outcomes [7]. 

In response to growing concerns about aesthetics, prefabricated zirconia crowns (ZCs) have been successfully introduced to pediatric dentistry through technological advancements. Two key advantages of ZCs are their outstanding esthetics and remarkable durability, while their highly polished surface has been shown to reduce plaque accumulation compared to other materials [8]. 

Numerous clinical trials have reported success rates of nearly 100% for zirconia crowns in primary dentition, and they are generally well-received by children and their parents for their aesthetic appeal. However, ZCs present clinical limitations, including high costs and the need for significant tooth reduction [9]. While zirconia crowns offer excellent aesthetics and high parent satisfaction, SSCs excel in crown retention and require less time and tooth preparation [10]. 

The newly introduced Bioflx crowns are esthetic crowns that require tooth preparation similar to traditional SSCs. Bioflx crowns are the first pediatric crowns designed to be flexible, durable, self-adaptable, and highly esthetic. They are composed of a high-impact hybrid radiopaque polymer resin, a material commonly used in medical equipment requiring exceptional strength, flexibility, and durability. These crowns are free from metal and bisphenol A-glycidyl methacrylate (BPA-GMA), making them a biocompatible option for pediatric dental restorations. Additionally, their monochromatic tooth-colored design allows them to effectively mask discoloration from arrested caries, enhancing their aesthetic appeal [11]. 

Despite the widespread use of crowns in pediatric dentistry, there is limited literature available on the properties of Bioflx crowns and their impact on clinical outcomes compared to conventional crown options.

This study provides valuable clinical insights into the comparative performance of three different crown types on primary molars by assessing plaque accumulation, debonding rate, and gingival health. The findings will contribute to evidence-based decision-making in pediatric dentistry, guiding clinicians in selecting the most effective crown type with optimal biocompatibility and long-term stability. Ultimately, this research has the potential to improve restorative strategies, promote better oral health outcomes, and inform future developments in pediatric dental materials and techniques.

This study aims to evaluate the clinical performance of stainless-steel, zirconia, and Bioflx crowns in pediatric patients. Additionally, it seeks to address existing knowledge gaps by systematically evaluating the clinical performance of three different crown types in primary molars.

This study tests the null hypothesis that there is no significant difference among the three different types of crowns placed on primary molars in terms of plaque index, crown debonding, loss of substance and gingival health.

## Materials and methods

### Protocol registration and study design

The datasets generated and analyzed during the current study are available at ClinicalTrials.gov under the identifier (NCT06706167). The study registration dates, first submitted on 23-11-2024, first submitted that met QC criteria on 23-11-2024 and first posted on 26-11-2024.

This study was approved by the Ethical Committee of the Faculty of Dentistry at Delta University for Science and Technology with registration number 0240721024. All procedures were in accordance with the ethical standards of the Helsinki Declaration of 1975, as revised in 2000.

The study procedures, including potential discomforts and risks, were explained to the parents of the participants, who provided informed written consent before the study commenced at the Pediatric Dentistry Department of Delta University for Science and Technology in Egypt.

**Fig. 1:**
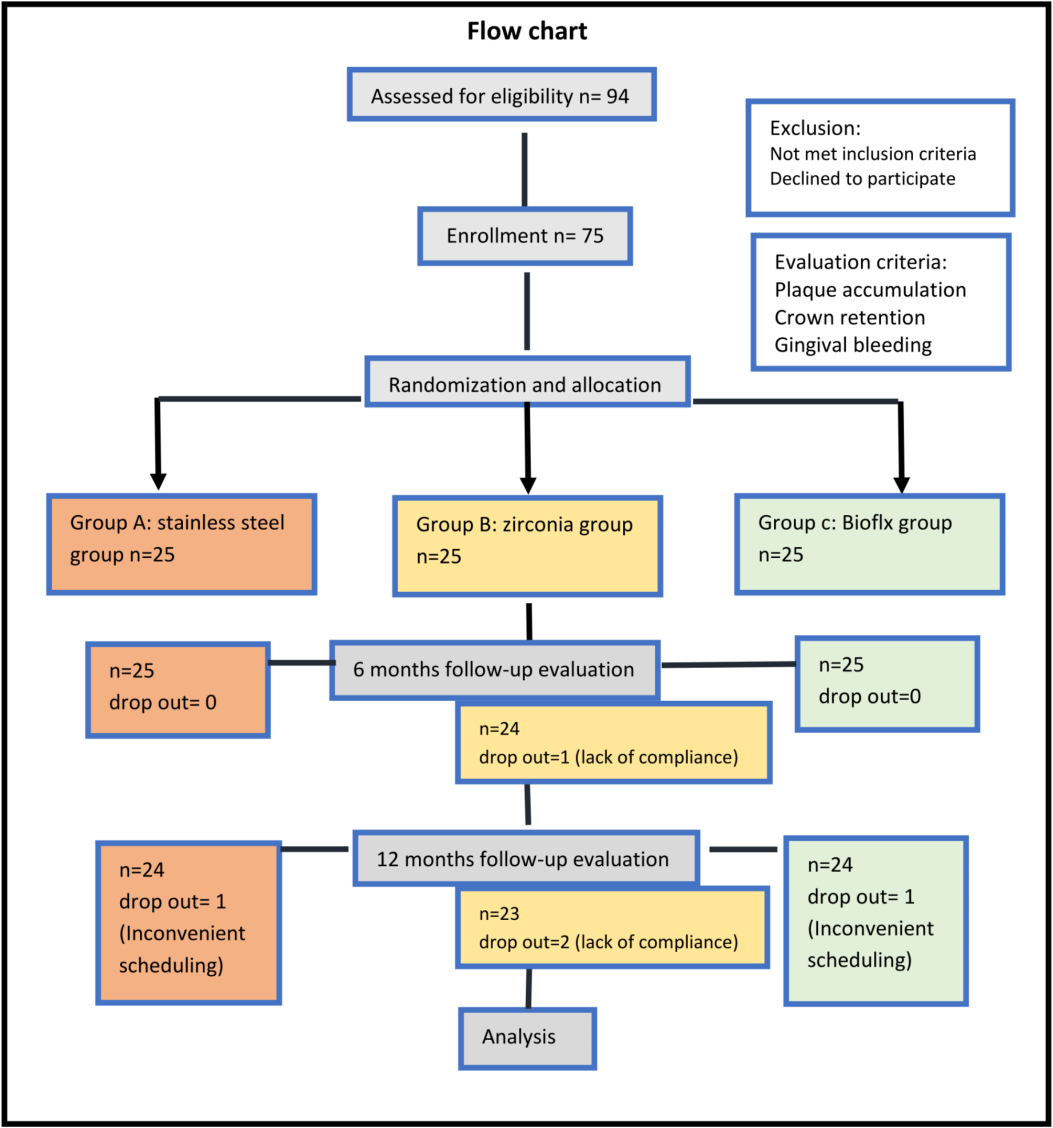
Flow diagram of study participants

This research was designed as a parallel randomized controlled trial conducted at Delta University for Science and Technology, Egypt, from September 2023 to September 2024. All procedures were performed by a single operator to minimize operator-induced errors. This trial was designed and reported following the CONSORT 2010 guidelines for randomized trials. The CONSORT flow diagram of the study participants is presented in Figure (1).

The sample size was calculated to achieve a 95% confidence level with a significance level set at *p* = 0.05. Assuming an estimated population of 500,000. To account for the finite population size, the sample size was adjusted using the finite population correction:

$$\:n=\frac{n0\bullet\:N}{N+n0-1}$$

where *N* = 500,000 is the total population size. This adjustment reduced the required sample size to 75 molars. To allow for potential dropouts, a final sample size of 75 was retained. These samples were evenly distributed into three groups of 25 molars each.

The selected sample size ensures adequate statistical power (80%) to detect clinically relevant differences between groups. This distribution supports the study’s aim to compare outcomes across multiple experimental treatments while maintaining sufficient statistical rigor.

Participants included healthy children aged 3 to 8 years, free of systemic diseases, with either pulpotomized or pulpectomized lower second primary molars, who exhibited a “definitely positive” or “positive” behavioral rating according to the Frankl scale.

Randomization was conducted using a sequence generation procedure from www.randomizer.org by principal investigator. Participants were divided into three groups: Group A received stainless steel crowns (control group), Group B received zirconia crowns (intervention group), and Group C received Bioflex crowns (intervention group). The allocation sequence was created prior to participant enrollment to ensure allocation concealment. The principal investigator enrolled participants and assigned them to intervention groups according to the predefined randomization sequence. The data analyst was blinded to the treatment groups to minimize bias in data interpretation, and participants in Group B (zirconia crown) and Group C (Bioflx crown) were blinded while participants in Group A (stainless steel crown) could not be blinded due to the metallic appearance of the crown.

### Clinical procedures

On the first visit, a brief medical and dental history was obtained. After performing pulpotomy or pulpectomy on the indicated tooth, a glass ionomer cement (GIC) (GC Gold, JAPAN) was placed as a core material. Local anesthesia was administered using 2% lidocaine with epinephrine at 1:100,000 prior to tooth preparation.

In all three groups, occlusal reduction of 1 to 1.5 mm was performed using a pear-shaped bur to ensure uniformity. Interocclusal clearance was checked using a wax sheet measuring 1.5–2 mm. Interproximal preparation was performed using a fine tapered diamond bur, and the proximal reduction was verified by passing a probe through the contact area. In the zirconia crown (NuSmile, USA) group (Group B), buccal and lingual preparation was required to be reduced by 0.5 to 1.25 mm as necessary to allow for a passive crown fit, with preparation extending subgingivally by 1 to 2 mm to eliminate any undercuts. All line and point angles were rounded to facilitate proper crown placement.

In the three groups, stainless Steel Crown (3 M ESPE, USA) (Group A), zirconia Crown (Group B), and Bioflx Crown (NuSmile, USA) (Group C) the crowns were selected based on the mesiodistal width of the prepared teeth. A trial fit was conducted before cementation in Groups A and C, whereas, a pink crown was used for the trial fit in Group B. After the crown trial and occlusion check, participants were instructed to bite into maximum intercuspation to ensure canine-to-canine contact. The prepared teeth were confirmed to be free of blood, saliva, and other residues, and the crowns were luted using GIC (GC Gold, JAPAN).

### Follow-up and evaluation criteria

Crown evaluations were conducted on the day of cementation and after one week for postoperative pain using the visual analog scale Wong Baker FACES [12], as well as through a questionnaire or phone interviews to assess any complaints about chewing problems. Additional evaluations at 6- and 12- months post-cementation included:

- **Plaque Accumulation**: This was measured using the Löe and Silness index, where each of the four surfaces of the crown (buccal, lingual, mesial, and distal) was scored from 0 to 3 (0 = no plaque, 3 = abundance of soft matter). The scores from the four areas were added and then divided by four to obtain the plaque index [10].
- **Crown debonding and substance loss**: Assessed using the USPHS criteria, where “Alpha” indicated the crown was continuous with the existing anatomic form, “Bravo” indicated a missing part of the crown but not enough to expose the underlying dentin/base, and “Charlie” indicated sufficient loss of crown substance to expose the underlying dentin/base or complete loss of the crown [13].
- **Gingival Health**: Evaluated using the bleeding index, with scores of 0 (no inflammation), 1 (mild inflammation), 2 (moderate inflammation), and 3 (severe inflammation) [14].

### Data analysis plan

Data were collected and analyzed statistically. Outcomes for each treatment were determined and compared using the Kruskal-Wallis H test, Friedman test and Chi-square test, as appropriate.

## Results

### Postoperative pain

A Kruskal-Wallis H test was conducted to examine the differences in post-operative pain scores. As shown in Table (1), a comparison of postoperative pain levels among the three different types of crowns, assessed using the Wong Baker scale after one week, revealed no statistically significant differences between the groups which (*p* = 0.926). This indicates that all crown types resulted in similar levels of postoperative discomfort.

**Table 1:** Wong baker scale mean pain scores

Crown types	Mean pain score	Median pain score	Standard deviation	Range (Min-Max)
Stainless steel crown	1.52	1	1.66	0–5
Zirconia crown	1.28	1	1.43	0–4
Bioflx crown	1.24	1	1.30	0–4

(*p* > 0.05) no statistically significant difference between SSC, ZC and Bioflx crown

### Plaque accumulation

#### At the 6 month follow up

As The Kruksal-Wallis H test was conducted to compare the median plaque index scores among the three types of crowns (Group A, Group B, and Group C). Plaque accumulation was minimal across all crown types, with no significant differences between stainless steel crowns (SSC), zirconia crowns, and Bioflx crowns (*P* = 0.709) as shown in Table (2).

#### At the 12 month follow up

The tabulated data in Table (2) demonstrated that plaque accumulation increased in all groups, with the highest median observed in the SSC group (0.5 [0–1]), followed by Bioflx (0.357 [0-0.875]) and zirconia (0.25 [0-0.5]), though the differences remained statistically insignificant (*P* = 0.578). Despite this, the within-group comparisons showed a significant increase in plaque accumulation over time for each crown type (*P* < 0.001), indicating that while plaque accumulation progressed over 12 months, no single crown type demonstrated a significantly higher tendency for plaque retention.

**Table 2:** Median scores of plaque index

Crown type Follow up	SSC Median (quartile range)	Zirconia crown Median (quartile range)	Bioflex crown Median (quartile range)	Test of significance *P* value*
Baseline	0(0–0)	0 (0–0)	0 (0–0)	1.000
6 months	0.25(0-0.25)	0 (0-0.25)	0.25 (0-0.25)	0.709
12 months	0.5(0–1)	0.25 (0-0.5)	0.357 (0-0.875)	0.578
*P* value**	**< 0.001**	**< 0.001**	**< 0.001**	-

*P* < 0.05 is statistically significant, *p* value*: Analysis done by Kruskal-Wallis H test, comparing three groups at each time point. *p* value**: analysis done by Friedman test to compare overtime changes in each group

### Crown debonding and loss of substance

#### At the 6- month follow-up

**Fig. 2:**
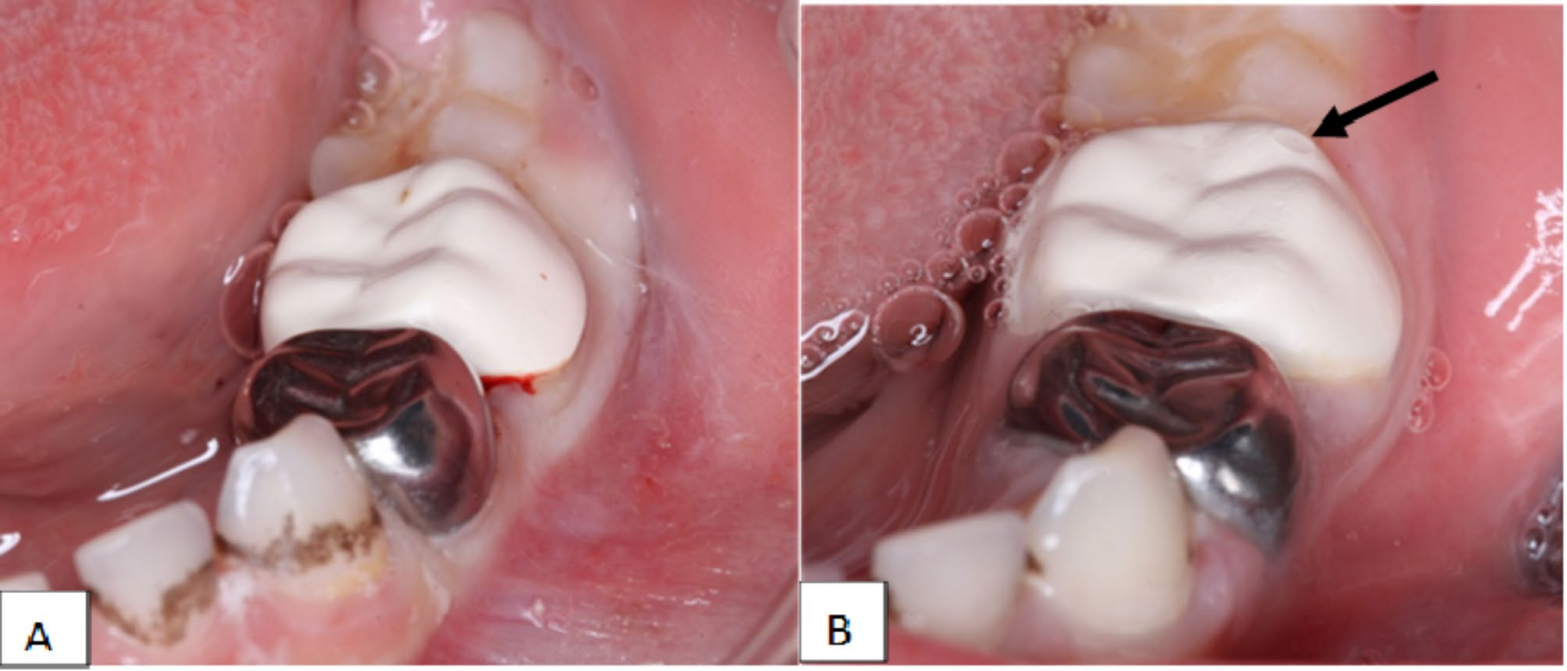
**A** showed no loss of material at baseline in Group C; Bioflx crown. **B** showed BRAVO score regarding retention at 6-month follow- up in group C; Bioflx crown, which black arrow indicate area of substance loss

As shown in Table (3), the Chi-Square Test of Independence was performed to examine the relationship between crown type and retention durability category. The results showed a Pearson Chi-Square statistic with a *p*-value of 0.425 and a Likelihood Ratio statistic with a *p*-value of 0.306. Since both *p*-values are greater than the significance level of 0.05, this suggests that there is no statistically significant association between crown type and retention durability category. In other words, the distribution of retention durability categories does not differ significantly among the various types of crowns. Additionally, as illustrated in Fig. 2A and B, Bioflx crowns Showed the highest score value of crown substance loss (BRAVO) at 6 months.

#### At the 12- month follow-up

Stainless Steel Crowns tended to have marginally better retention rates compared to zirconia and Bioflx; however, the data presented in Table (3) suggest that the difference was not statistically significant. The Chi-Square Test of Independence was performed to examine the relationship between crown type and retention durability category. The results showed a Pearson Chi-Square statistic with a *p*-value of 0.611 and a Likelihood Ratio statistic with a *p*-value of 0.520. Since both *p*-values are greater than the significance level of 0.05, this suggests that there is no statistically significant association between crown type and retention durability category. In other words, the distribution of retention durability categories does not differ significantly among the various types of crowns. Additionally, as illustrated in Table 3, zirconia crowns showed the highest score value of crown debonding (CHARLIE) at 12 months while, SSCs revealed the lowest.

**Table 3:** Comparison between SSC, Zirconia crown and Bioflx crown in terms of retention

Time Crown type	6 months				12 months			
	Alpha (*n*%)	Bravo (*n*%)	Charlie (*n*%)	Completed (*n*%)	Alpha (*n*%)	Bravo (*n*%)	Charlie (*n*%)	Completed (*n*%)
SSC	24(96%)	1 (4%)	0 (0%)	25 (100%)	22(91.6%)	1(4.1%)	1(4.1%)	24 (96%)
Zirconia crown	23(95.8)	0 (0%)	1(4.1%)	24 (96%)	20(86.9%)	0 (0%)	3 (13%)	23 (92%)
Bioflx crown	23(92%)	2 (8%)	0 (0%)	25 (100%)	21(87.5%)	2(8.3%)	1(4.2%)	24 (96%)
Total	70(94.5%)	3(4%)	1(1.3%)	74	63(88.7%)	3(4.2%)	5(7%)	71

*Alpha = crown was continuous with the existing anatomic form*

*Bravo = missing part of the crown*

*Charlie = complete loss of crown*

### Gingival inflammation

#### At the 6 month follow up

As shown in Table (4), the Kruskal-Wallis H Test was conducted to compare gingival inflammation scores across the different crown types where gingival inflammation remained minimal across all crown types, with median values ranging from 0 to 0.5. There was no statistically significant difference in gingival inflammation between stainless steel crowns (SSC), zirconia crowns, and Bioflx crowns (*P* = 0.770), indicating that all three materials had a similar impact on gingival health at this stage.

#### At the 12 month follow up

**Fig. 3:**
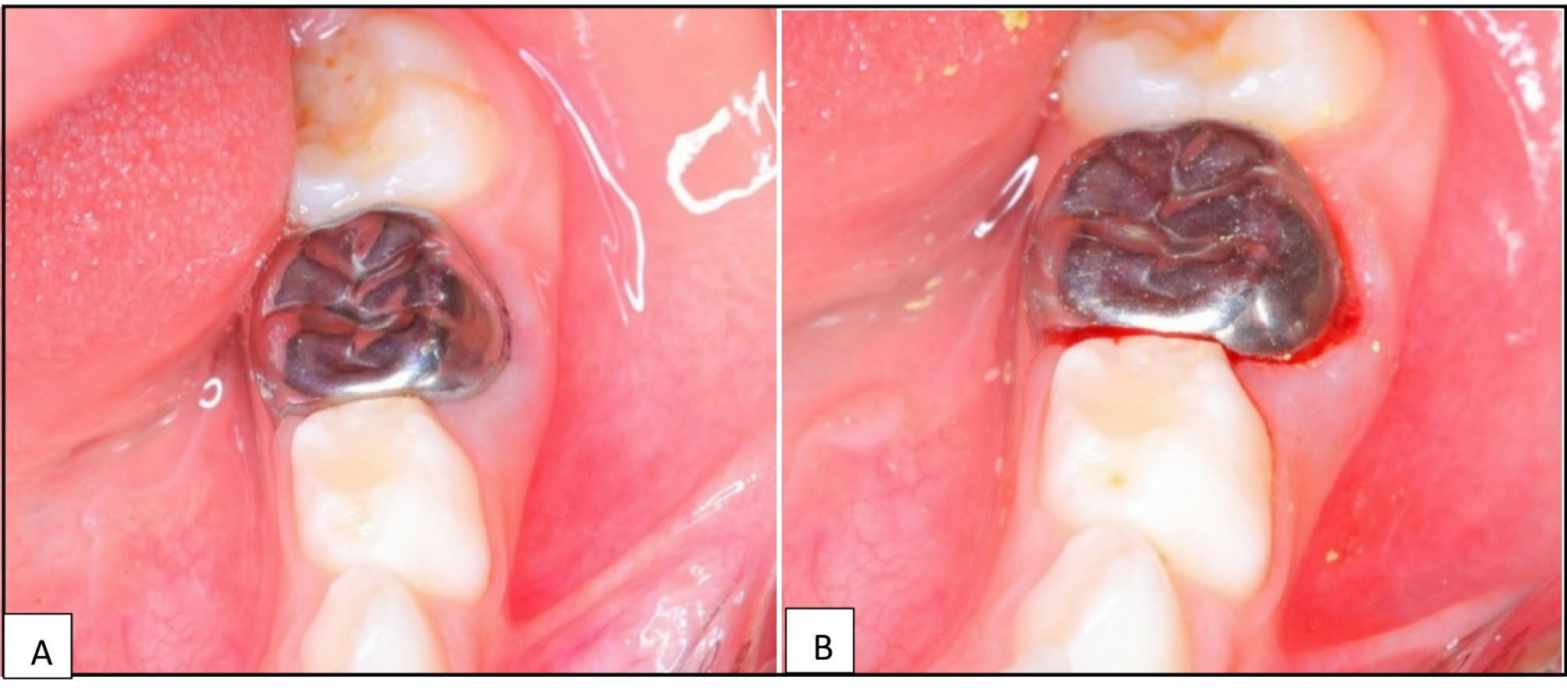
**A** and **B** showed the difference in gingival bleeding index at 6- and 12- month follow- ups in group A; stainless steel crown

Gingival inflammation had slightly increased in all groups, but the differences between crown types remained statistically insignificant (*P* = 0.747). However, within-group comparisons showed a significant increase in gingival inflammation over time for each crown type (*P* < 0.001), suggesting that while gingival inflammation progresses over time, it is not significantly influenced by the type of crown used. Furthermore, stainless steel crown exhibited the highest mean value among all groups, whilst zirconia crown revealed the lowest. Figure 3A and B showed the difference in gingival bleeding index in Group A; stainless-steel crown group at 6 and 12 month follow up.

**Table 4:** Median scores of gingival indices among three groups

Crown type Follow up	SSC Median (quartile range)	Zirconia crown Median (quartile range)	Bioflex crown Median (quartile range)	Test of significance *P* value*
Baseline	0(0–0)	0 (0–0)	0 (0–0)	1.000
6 months	0(0-0.5)	0 (0-0.25)	0 (0-0.5)	0.770
12 months	0(0–1)	0 (0-0.5)	0. (0-0.75)	0.747
*P* value**	**< 0.001**	**< 0.001**	**< 0.001**	-

*P* < 0.05 is statistically significant, *p* value*: Analysis done by Kruskal-Wallis H test, comparing three groups at each time point. *p* value**: analysis done by Friedman test to compare overtime changes in each group

## Discussion

As dental professionals continue to seek optimal solutions for restoring primary teeth, this study aims to compare clinical outcomes, specifically, plaque accumulation, gingival health, and crown debonding of three different types of prefabricated primary crown; stainless steel crowns, zirconia crowns, and Bioflx crowns.

The findings of this study indicate that stainless steel, zirconia, and Bioflx crowns showed comparable outcomes in terms of plaque accumulation, debonding rate, substance loss, and gingival bleeding, with no statistically significant differences observed, which the results did not provide sufficient evidence to reject the null hypothesis.

The random allocation ensured an even distribution of patient-related factors (such as oral hygiene and occlusion) across the groups, reinforcing the reliability of the comparative outcomes for plaque accumulation, gingival health, and crown retention.

The standardization of preparation and cementation likely contributed to the results across all groups. Since all crowns were seated under identical conditions, any variations in plaque retention would be primarily influenced by material properties rather than operator-dependent factors.

Blinding the data analyst was crucial in reducing potential observer bias when recording plaque accumulation, gingival health, crown retention and loss of substance. Since the evaluator was unaware of the treatment assignment, the risk of subconsciously favoring one crown type over another was minimized. However, complete blinding of the clinical operator was not possible, as they had to perform different preparation techniques for each crown type.

This study is the first to evaluate postoperative pain following the cementation of different crown types in primary molars. Our findings revealed no statistically significant differences in postoperative pain between the groups, suggesting that the type of crown cemented may not influence pain perception in pediatric patients. While direct comparisons with previous studies are not possible due to the novelty of this research. These findings highlight the importance of continued exploration into material biocompatibility and postoperative outcomes. Future studies could examine long-term outcomes and pain in larger, more diverse populations to validate these preliminary findings.

In terms of plaque accumulation and gingival inflammation, while no significant differences were observed among the three crown types, zirconia crowns demonstrated the lowest scores compared to stainless steel (SSC) and Bioflx crowns. The results are in congruence with a study done by Taran and Kaya in 2018 which indicated that zirconia crowns had better plaque index (PI) and gingival index (GI) scores relative to control teeth [15]. Previous studies by Beldüz Kara and Yilmaz (2014) also reported an association between SSCs and gingivitis [16]. Another study by Mathew et al. (2020) evaluated the clinical outcomes of bilateral pulp therapy in patients fitted with either zirconia or stainless steel crowns. Although no statistical differences were found in overall success rates for the two crown types, less plaque accumulation was noted with zirconia, and both crown types received high parental satisfaction ratings [17]. This advantage may be attributed to the smooth surface of zirconia, which minimizes plaque accumulation [18]. On the other hand, SSCs require extensive manipulation, including cutting, crimping, and trimming, to achieve an accurate marginal fit. This process can introduce surface defects and roughness, facilitating plaque buildup and microbial attachment. Furthermore, these manipulations can distort the metal substructure or lead to the release of metal ions at the gingival margin, concerns that are less relevant for zirconia crowns.

Conversely, Agrawal et al. (2022) reported higher plaque accumulation on zirconia crowns than on stainless steel crowns, which could be attributed to the participants’ poor oral hygiene and the study’s short follow-up duration [10]. 

Regarding debonding rate and loss of crown substance, there was no statistical difference among different crown types. However, stainless steel and Bioflx crowns showed better results regarding debonding rate of crown than zirconia crown at 6- and 12-month follow-up. These results came in agreement with Argawal et al. (2022) which stainless steel crown showed better results regarding retention of crown than zirconia at 3rd month follow-up [10]. This trend alignment could be due to the passive fit of zirconia crowns so they largely depend on cements for retention. However, these findings do not conform to findings of Abdulhadi et al. who reported 100% retention of zirconia crowns when cemented with resin cement or resin-modified glass ionomer cement which contradict with the current study that used GLC for cementation [19]. The additional buccal and lingual reduction required for zirconia crowns may explain their slightly higher debonding rate, as their retention depends largely on cement adhesion rather than mechanical retention, unlike SSCs and Bioflx crowns, which can be crimped for a tighter fit.

This study provides meaningful insights into the performance of crown materials under well-controlled conditions. The results offer a strong foundation for understanding material behavior and serve as a valuable guide for clinical decision-making. While the study was conducted on a specific sample size and population, the controlled design ensures that the findings are reliable and reproducible within similar contexts. Moreover, the inclusion criteria enhance the applicability of the results to a wide range of clinical scenarios. Additionally, the study offers an important step toward optimizing crown material selection.

This study has several limitations that should be considered:

1. One limitation of this study is the relatively small sample size.
2. The follow-up period in this study was limited, which may not fully capture the long-term performance.
3. Variables such as age, oral hygiene habits, occlusal forces, and dietary influences were not fully accounted for in this study. These factors can significantly impact the clinical performance of different crown materials.
4. This study did not evaluate patient- or parent-reported outcomes, which are essential for understanding comfort, esthetic preferences, and overall satisfaction with different crown materials.

Based on the findings of this study, several recommendations can be made for future research and clinical practice:

Firstly, longer-term follow-up is necessary to confirm these outcomes and provide more robust conclusions regarding the clinical performance of stainless steel, zirconia, and Bioflx crowns.

Secondly, a larger sample size would help determine whether the observed similarities in plaque accumulation, gingival inflammation, debonding rates, and substance loss remain consistent, and whether any differences between the materials reach statistical significance.

Thirdly, factors such as individual oral hygiene habits and dietary influences on different crown materials may play a significant role in clinical success and should be considered in future studies.

Lastly, patient and parent satisfaction should be a key consideration in future research and clinical decision-making.

## Conclusions

1. The differences in plaque accumulation, gingival health, and crown retention among various types of crowns are not clinically significant to mandate the use of one type over another. Consequently, clinical expertise and individual patient factors are likely to play an important role in crown selection.
2. Plaque accumulation and gingival inflammation significantly increased in all crown types after six months, indicating a progressive rise over time regardless of the material used, emphasizing the critical role of patient hygiene in maintaining oral health.
3. Since postoperative pain was comparable across all crown types, selection should be based on clinical needs, esthetics, and patient preference.
4. Clinically, zirconia crowns offer better gingival health and lower plaque accumulation, making them ideal for patients prone to inflammation. In contrast, stainless steel and Bioflx crowns provide superior retention, making them more reliable for long-term stability.

## Electronic supplementary material

Below is the link to the electronic supplementary material.

Supplementary Material 1

Supplementary Material 2

## Data Availability

The datasets generated and analyzed during the current study are available at ClinicalTrials.gov under the identifier NCT06706167, data related to the study’s protocol, methodology, and results are accessible on the registry with registration date on 26-11-2024.
